# An Electric Instrument Shop

**Published:** 1906-11

**Authors:** 


### An Electric Instrument Shop.

UNTIL recently one of the greatest drawbacks to the satisfactory use of electro-therapeutic instruments in Buffalo, has been the inconvenience to which physicians have been put of having to send out of town for parts and repairs. However slight the break the whole instrument must be sent to another city to be placed in workable condition. Even when sent to local repair shops of a purely electric character the work done was hardly satisfactory because of the lack of the special technical knowledge required in the upbuilding of the modern electric lighted instruments. The busy physician using instruments or appliances of an electric type need wait no longer now, than the time necessary for the passage of a telephone message to place his defective instrument in the hands of an expert, since the Buffalo Electro-Mechanical Company has begun business at 83 West Chippewa street. The experts employed here are thoroughly conversant with every intricacy of all electrotherapeutic apparatus; they suggest methods of betterment in the use and care of such instruments so that the best result with the least destruction may be secured. Not alone that, but they make instruments from the very foundation. The company is in constant touch with electrotherapeutic advance and every part of all the standard appliances is in stock and can be supplied at a moment's notice; all the standard lamps from the smallest to the largest used, and of every type, may be had by special delivery if necessary for the trouble of telephoning.

Physicians using electrotherapeutic instruments and apparatus may be assured of immediate service in the future, by asking that one of the company's experts be sent to his office to look at his outfit and make record of parts and sizes which it may be necessary to replace, so that an order may be filled without delay. For this inspection and recording of sizes of lamps and parts no charge will be made. All that is necessary after that is a telephonic order and the required part will be delivered at once. The company does not confine its electric activities to therapeutics; it has special experts in all other branches of electric work. A useful instrument which the company will soon put on the market is a small diagnostic lamp for office work illuminated by dry cell batteries.

---

THE trained nurse idea has caught on tremendously in France (*The Tribune,* October 17), where at last a training school has been opened. Hitherto the French nurse has been a person to be dreaded. Either she was a "Sairey Gamp," with no more medical knowledge than she could tuck away in her apron pocket, or she was a Sister of Mercy, trained in a convent with a view more to the healing of souls than of bodies. The nurse à l'Anglaise was a capable, businesslike person, with a wholesome respect for herself and her profession and a practical skill in excess of many a country physician. To know her was to approve, and the French, with their good practical sense, have decided to duplicate her. Hence the new training school at Bordeaux, where the nurses—well housed and well instructed—are under the supervision of an Englishwoman, a Miss Elston, who has been trained in the London hospitals. The work seems to appeal to the better class of French girls of good bourgeois stock and with a lycée training. The French nurse, new style, will soon be seen in every hospital in France.

---

It is unnecessary to present any argument to well informed physicians or to educated dentists in regard to the advantages of oxygen as a germ destroying agent. Pure air is always well laden with oxygen which is the greatest purifier in nature's laboratory. Chemists in recent years have given us a combination of oxygen and lime which has been named "Calox" and which is the greatest mouth purifier that has ever been offered to the people. Every person of refinement desires to preserve the teeth. Calox not only sweetens the mouth but it prevents decay through destruction of putrefactive germs. Anyone who desires to test the efficacy of Calox as a dentifrice should send for a sample to McKesson & Robbins, 91 Fulton street, New York.

---

"The Practitioner" sounds a note of warning (*The Tribune,* October 18) against the dangers of the strenuous life: It is good to be strenuous, but it is also good, as the poet tells us, to play the fool, or at any rate to be idle at the right time and in the right way. This is just what the strenuous man forgets, and the consequence is too often premature breakdown,—a common event in the storm and stress of modern life. The strenuous life is helping to overcrowd our asylums. This, in "The Practitioner's" view, consists not in a change of excitement or in hard work disguised as a game, but in that "genuine repose of which Charles James Fox, strenuous as he was in politics and in play, was thinking when he said there was nothing so pleasant as to lie under a shady tree with a book except to do so without a book."

